# BLACK AND WHITE YOUNG ADULTS’ SUPPORT TO MIDLIFE PARENTS

**DOI:** 10.1093/geroni/igz038.1050

**Published:** 2019-11-08

**Authors:** Kristie A Wood, Meng Huo, Yee To Ng, Karen Fingerman

**Affiliations:** University of Texas, Austin, Austin, Texas, United States

## Abstract

Researchers have observed racial differences in support to midlife parents. Black adults typically provide more support to parents and report greater rewards in doing so. We were interested in whether this differential trend can be observed in young adulthood. Furthermore, we aimed to understand cultural beliefs underlying any racial differences in support provided to parents. We examined support Black and White young adults provided to their parents, and beliefs associated with that support. Young adults (aged 18–35 years; 26%, n=184 Black and 74%, n=525 White) from the Family Exchanges Study II (2013) participated. They reported how often they provided 4 types of support (financial, technical, practical, emotional) to each parent (N =824) on a scale from 1 = once a year or less often to 8 = everyday. Multilevel models revealed Black young adults provided more frequent support to parents than White young adults, mediated by beliefs about familial obligation. Interestingly, we also found that Black young adults report significantly more negative relationship quality with parents and a stronger desire for support from parents when compared to White young adults. Findings suggest that Black young adults may espouse collective and interdependent values such as the ability to provide for a family and to receive support from family. Further, more frequent support may co-occur with conflicts that suggest that congruence between values and support do not necessarily inspire harmonious ties.

